# Magnetic Resonance Imaging Utilization in an Emergency Department Observation Unit

**DOI:** 10.5811/westjem.2017.6.33992

**Published:** 2017-07-19

**Authors:** Yadiel Sánchez, Brian J. Yun, Anand M. Prabhakar, McKinley Glover, Benjamin A. White, Theodore I. Benzer, Ali S. Raja

**Affiliations:** * Harvard Medical School, Massachusetts General Hospital, Department of Radiology, Boston, Massachusetts; † Harvard Medical School, Massachusetts General Hospital, Department of Emergency Medicine, Boston, Massachusetts; ‡ Center for Research in Emergency Department Operations (CREDO), Massachusetts General Hospital, Department of Emergency Medicine, Boston, Massachusetts; § Harvard Medical School, Massachusetts General Hospital, Department of Radiology, Division of Emergency Imaging, Boston, Massachusetts; ¶ Massachusetts General Physicians Organization, Massachusetts General Hospital, Boston, Massachusetts

## Abstract

**Introduction:**

Emergency department observation units (EDOUs) are a valuable alternative to inpatient admissions for ED patients needing extended care. However, while the use of advanced imaging is becoming more common in the ED, there are no studies characterizing the use of magnetic resonance imaging (MRI) examinations in the EDOU.

**Methods:**

This institutional review board-approved, retrospective study was performed at a 999-bed quaternary care academic Level I adult and pediatric trauma center, with approximately 114,000 ED visits annually and a 32-bed adult EDOU. We retrospectively reviewed the EDOU patient database for all MRI examinations done from October 1, 2013, to September 30, 2015. We sought to describe the most frequent uses for MRI during EDOU admissions and reviewed EDOU length of stay (LOS) to determine whether the use of MRI was associated with any change in LOS.

**Results:**

A total of 22,840 EDOU admissions were recorded during the two-year study period, and 4,437 (19%) of these patients had a least one MRI examination during their stay; 2,730 (62%) of these studies were of the brain, head, or neck, and an additional 1,392 (31%) were of the spine. There was no significant difference between the median LOS of admissions in which an MRI study was performed (17.5 hours) and the median LOS (17.7 hours) of admissions in which an MRI study was not performed [p=0.33].

**Conclusion:**

Neuroimaging makes up the clear majority of MRI examinations from our EDOU, and the use of MRI does not appear to prolong EDOU LOS. Future work should focus on the appropriateness of these MRI examinations to determine potential resource and cost savings.

## INTRODUCTION

Although most patients presenting to an emergency department (ED) will subsequently be discharged,^1^ many patients with more serious conditions will require admission to the hospital for further evaluation and management. Within the Medicare population, hospital inpatient services represent a significant portion of overall payments for beneficiaries, which has led to increased efforts aimed at enhancing both the value and quality of care delivered.^2^

One solution for optimizing care delivery has been the development and utilization of emergency department observation units (EDOU), a potential disposition option for patients who do not meet the criteria for inpatient admission but who cannot be discharged without additional care.^3^–^7^ More than two million U.S. EDOU admissions were reported in 2011 alone.^1^ Observation units provide clinicians additional time to either provide care or order diagnostic testing that can direct further patient management.^8^ EDOUs have been shown to reduce overall hospital costs,^5^,^9^ and it is estimated that they may save more than $3.0 billion annually in the U.S.^10^ Baugh et al. found that using protocols in EDOUs for patients who present with syncope could save more than $100 million annually at a national level.^5^ Although it is estimated that only one-third of EDs in the U. S. have an EDOU,^6^–^7^ the ratio of EDOU stays to inpatient admissions has been rising.^11^

Diagnostic imaging is a critical component of care in both the ED and the EDOU, with nearly half of all ED visits in the U.S resulting in at least one imaging examination in 2011.^1^ A 2015 study found that patients admitted to an EDOU were more likely to undergo magnetic resonance imaging (MRI) than those who were admitted as inpatients.^4^ However, while the use of advanced imaging is becoming more common in the ED,^12^,^13^ little is known about the utilization of MRI in EDOUs. Therefore, the aim of this study was to characterize the frequency of MRI examinations performed on patients in an EDOU, stratified by anatomical area. The secondary objective was to determine if MRI exam performance affected the length of stay (LOS) in the EDOU.

## METHODS

### Human Subjects Compliance

This retrospective descriptive Health Insurance Portability and Accountability Act–compliant study was approved by our institution’s institutional review board, including a waiver of patient consent.

### Study Site

The study was performed at a 999-bed quaternary care academic Level I adult and pediatric trauma center, with approximately 114,000 ED visits annually. Approximately 105,000 ED and EDOU diagnostic imaging studies are performed and interpreted by the division of emergency radiology annually. The EDOU is composed of a 32-bed observation unit with emergency physician supervision and receives over 11,000 admissions annually.

Population Health Research Capsule

What do we already know about this issue?

More EDs are placing patients in observation units instead of admitting them to the hospital, and some of these patients need MRIs.

What was the research question?

How are MRIs being used in an academic ED’s observation unit, and are they adding to the length of stay?

What was the major finding of the study?

Approximately one-fifth of patients had an MRI, and these patients did not have a longer length of stay.

How does this improve population health?

MRIs are regularly performed in observation units, and we should focus on determining which MRIs are appropriate and which can be done as outpatient tests instead.

### Collection of Patient Data

The study period was from October 1, 2013, to September 30, 2015. We retrospectively retrieved data from the hospital reporting system, including all MRI studies performed in the EDOU. These studies were characterized by anatomical area using the exam description. We also obtained the LOS for each admission in the EDOU, defined as the time elapsed in hours between the patient’s admission into the EDOU and their subsequent discharge from the unit.

### Outcome Measures

The primary outcome measures for this study were the overall proportion of EDOU admissions that included an MRI examination (MRI utilization), as well as the distribution of these examinations by anatomical area. The secondary outcome measure compared the median EDOU LOS of patients with and without MRI examinations.

### Statistical Analysis

Data was imported into Microsoft Excel (Redmond, WA) for further analysis. We used summary statistics to describe overall MRI utilization and MRI distribution by anatomical area.

We performed a two-tailed, Wilcoxon rank-sum test between the median of EDOU LOS for admissions with and without a MRI study. Statistical significance was set at p<0.05.

## RESULTS

### MRI Utilization and Distribution

A total of 22,840 EDOU admissions were recorded during the two-year study period. Among these admissions, 4,437 (19%) included at least one MRI examination. The overall distribution of these exams is depicted in Table 1. The most common exam was MRI of the brain, head, or neck, conducted in 2,730 (62%) examinations, followed by MRI exam of the spine, performed in 1,392 (31%) examinations (Table 2). The MRI examination distribution of the musculoskeletal system and abdomen/genitourinary area is presented in Table 3.

### EDOU Length of Stay (LOS)

There was no LOS information on five admissions where an MRI study was not performed (0.1%), and these admissions were excluded from this analysis. There was no significant difference between the median LOS of admissions where an MRI study was performed (17.5 hours) and the median LOS (17.7 hours) of admissions where an MRI study was not performed [p=0.33].

## DISCUSSION

In the spectrum of clinical care, EDOUs represent a valuable alternative to inpatient admissions. Previous authors have noted that patients in the EDOU are more likely to undergo MRI examination when compared to those admitted to an inpatient service.^4^ In this study, we assessed the utilization and distribution of MRI studies performed at one of the largest EDOUs in the U.S. Several of our findings are of interest.

The greatest proportion (62%) of the MRI examinations performed in our EDOU population were studies of the brain, head, or neck. One reason for these findings may be that EDOUs have been shown to be cost-effective for evaluating acute neurologic conditions, specifically transient ischemic attacks (TIAs).^14^ Guidelines support use of MRI examinations for appropriate patients with symptoms of TIA.^15^ Further, hospitals seeking comprehensive stroke certification from The Joint Commission must have MRI scanner availability 24 hours/day, 7 days/week,^16^ highlighting the importance of advanced imaging in patients presenting with acute neurological symptoms. Our institution has specific protocols for patients who present with symptoms of a TIA that suggest they undergo MRI imaging in the EDOU. Having evidence-based protocols in an EDOU, specifically regarding which imaging is best performed in the EDOU and which may be safely performed in an outpatient setting, has been shown to lead to shorter hospital stays and lower overall costs.^17^

In addition, there was no significant difference in the LOS of EDOU admissions for patients with and without MRI examinations. Although we did not fully assess some of the factors associated with the LOS in observation units, including age, type of insurance, reason for EDOU admission, and others,^18^ the carefully designed protocols, available personnel, and robust imaging resources of our dedicated observation unit may in part explain the lack of variation in LOS for these patients. Our median LOS for patients who underwent MRI was less than half of the 48-hour limit suggested by the Centers for Medicare and Medicaid Services,^8^ suggesting that our EDOU was able to evaluate these patients in an efficient manner – potentially saving inpatient admissions without burdening our ED with prolonged patient work-ups.

## LIMITATIONS

This study has several limitations. First, it was a retrospective, single-institution study that may limit generalization of our findings to other institutions. Second, we did not assess patient demographics, patient chief complaint in the ED, or reason for ordering the MRI examination, all which may have influenced the pattern of distribution of MRI imaging and the EDOU LOS. Finally, we did not assess the clinical outcomes of the patients treated in the EDOU and those subsequently admitted to the hospital.

## CONCLUSION

In this study, neuroimaging made up the vast majority of MRI examinations from our EDOU, and patients in whom an MRI was performed did not have a longer LOS than those who did not. Future work should focus on the appropriateness of these MRI examinations to determine potential resource and cost savings.

## Figures and Tables

**Table 1:** Distribution of MRI examinations performed in the emergency department observation unit by anatomical area.

Anatomical area	N	%
Brain/head/neck	2730	61.5%
Spine	1392	31.4%
Musculoskeletal extremity	232	5.2%
Abdomen	47	1.1%
Pelvis	31	0.7%
Other*	5	0.1%
Total	4437	100.0%

* magnetic resonance angiography aortic arch, MRA upper extremity, 3 studies unknown.

**Table 2:** Distribution of MRI spine examinations performed in the emergency department observation unit.

Anatomical Area	N	%
Lumbar spine	726	52.2%
Cervical spine	363	26.1%
Thoracic spine	163	11.7%
Entire spine	128	9.2%
Sacrum	11	0.8%
MRA spine	1	0.1%
Total	1392	100.0%

*MRA,* magnetic resonance angiography; *MRI,* magnetic resonance imaging.

**Table 3:** Distribution of MRI examinations of the musculoskeletal system and abdomen/genitourinary area, performed in the emergency department observation unit.

Anatomical Area	N	%
Musculoskeletal		
Hip	89	38.4%
Pelvic bone	33	14.2%
Knee	29	12.5%
Foot	29	12.5%
Shoulder	14	6.0%
Leg	11	4.7%
Femur	10	4.3%
Brachial plexus	6	2.6%
Wrist	3	1.3%
Ankle	3	1.3%
Elbow	2	0.9%
Arm	1	0.4%
Humerus	1	0.4%
Hand	1	0.4%
Total	232	100.0%
Abdomen/genitourinary		
Pelvis	31	39.7%
MRCP	26	33.3%
Liver	9	11.5%
Enterography	6	7.7%
Kidney	2	2.6%
Pancreas	2	2.6%
Adrenal	1	1.3%
Rectum	1	1.3%
Total	78	100.0%

*MRI,* magnetic resonance imaging, *MCRP*, magnetic resonance cholangiopancreatography.

## References

[1] National Hospital Ambulatory Medical Care Survey 2011 Emergency Department Summary Tables.

[2] Medicare Payment Advisory Commission (2016). A data book: health care spending and the Medicare program.

[3] Baugh CW, Schuur JD (2013). Observation Care — High-Value Care or a Cost-Shifting Loophole?. N Eng J Med.

[4] Prabhakar AM, Misono AS, Harvey HB (2015). Imaging utilization from the ED: no difference between observation and admitted patients. Am J Emerg Med.

[5] Baugh CW, Liang LJ, Probst MA (2015). National cost savings from observation unit management of syncope. Acad Emerg Med.

[6] Wiler JL, Ross MA, Ginde AA (2011). National study of emergency department observation services. Acad Emerg Med.

[7] Venkatesh AK, Geisler BP, Gibson Chambers JJ (2011). Use of observation care in US emergency departments, 2001 to 2008. PLoS One.

[8] Centers for Medicare and Medicaid Services Medicare benefit policy manual: Chapter 6—hospital services covered under Part B.

[9] Abbass IM, Krause TM, Virani SS (2015). Revisiting the economic efficiencies of observation units. Manag Care.

[10] Baugh CW, Venkatesh AK, Hilton JA (2012). Making greater use of dedicated hospital observation units for many short-stay patients could save $3.1 billion a year. Health Aff (Millwood).

[11] Feng Z, Wright B, Mor V (2012). Sharp rise in Medicare enrollees being held in hospitals for observation raises concerns about causes and consequences. Health Aff (Millwood).

[12] Korley FK, Pham JC, Kirsch TD (2010). Use of advanced radiology during visits to US emergency departments for injury-related conditions, 1998–2007. JAMA.

[13] Bhuiya FA, Pitts SR, McCaig LF (2010). Emergency department visits for chest pain and abdominal pain: United States, 1999–2008. NCHS Data Brief.

[14] Ross MA, Compton S, Medado P (2007). An emergency department diagnostic protocol for patients with transient ischemic attack: a randomized controlled trial. Ann Emerg Med.

[15] Oostema JA, Delano M, Bhatt A (2014). Incorporating diffusion-weighted magnetic resonance imaging into an observation unit transient ischemic attack pathway: a prospective study. Neurohospitalist.

[16] The Joint Commission Advanced Certification Comprehensive Stroke Centers.

[17] Ross MA, Hockenberry JM, Mutter R (2013). Protocol-driven emergency department observation units offer savings, shorter stays, and reduced admissions. Health Aff (Millwood).

[18] Hockenberry JM, Mutter R, Barrett M (2014). Factors Associated with Prolonged Observation Services Stays and the Impact of Long Stays on Patient Cost. Health Serv Res.

